# European Training Assessment Programme 2.0

**DOI:** 10.1186/s13244-019-0797-4

**Published:** 2019-11-04

**Authors:** European Board of Radiology (EBR)

**Affiliations:** Barcelona, Spain

**Keywords:** Training, Certification, Assessment methods

## Abstract

The European Training Assessment Programme (ETAP) 2.0 offers a structured assessment of radiology training institutions applying for certification, without geographical constraints. It applies, in fact, to both European and non-European institutions, which fulfill the eligibility criteria and wish to obtain a European certification.

ETAP 2.0 aims to provide centres that offer specialty training in radiology with an objective assessment by external assessors of their training programmes. ETAP 2.0 also aims to develop assessment systems and guidelines to be used by postgraduate education authorities at a national level.

The online evaluation system facilitates the application process as well as the assessment and subsequent certification. The platform enables users – both representatives of applicant institutions and assessors – to easily and efficiently store, access, and manage documents and information at any time, thereby facilitating the certification process.

## Key Points

- The European Training Assessment Programme (ETAP) 2.0 offers a structured assessment of radiology training institutions.
- ETAP 2.0 represents a step forward in the attempt to harmonise radiological training around Europe
- The assessment is performed online. It is accurate, objective, fast, and cost-effective.
- ETAP 2.0 offers training centres an opportunity for self-development.

## Introduction

The European Training Assessment Programme (ETAP) was established as a joint initiative of the former European Association of Radiology (EAR) and the European Union of Medical Specialists (UEMS) Radiology section in 2001, with the aim of assessing and harmonising radiology training programmes of health institutions in Europe.

In March 2016, the ESR and the UEMS Radiology section agreed to renew the project and established the ETAP 2.0. A qualitative adaptation was made, and the programme’s structure transitioned from face-to-face audit to online audit in order to facilitate access for a larger number of centres and lighten the workload of the assessors. This was done to achieve recognition as a certificate of excellence and as an “added value” by different European and international institutions.

**Fig. 1:**
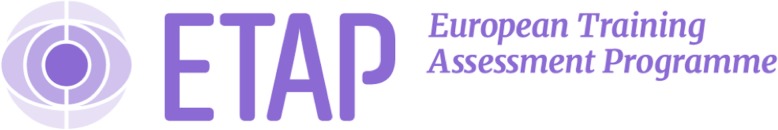
ETAP logo

At the same time, a decision was made to move the project under the umbrella of the European Board of Radiology (EBR), meaning that the EBR would oversee the programme in line with its objective of harmonising radiological standards in education. The ETAP 2.0 was successfully launched at the European Congress of Radiology (ECR) 2018 (Fig. 1).

ETAP 2.0 represents a step forward in the attempt to harmonise radiological training around Europe, which will be of major benefit to healthcare systems across-the-board [1]. The new assessment programme has been designed to be conducted online in order to make it more user-friendly for both assessors and training centres. Moreover, it is faster and more cost-effective for the ESR, the UEMS, and the assessed centre.

## Structure

The ETAP Scientific Committee responsible for the assessment consists of an equal number of EBR and UEMS members. Candidates from the EBR and the UEMS alternate as scientific directors. Three EBR members and three UEMS members fulfil assessor roles on a rotating basis (one lead assessor and co-assessor per assessed institution). Lastly, two junior assessors (European junior doctors and ESR Radiology Trainees Forum (RTF) Subcommittee representatives) hold an advisory role as the residents or junior doctors supporting the assessors. The ESR national delegates to the Education Committee as well as the national delegates to the UEMS Section of Radiology may serve as verifiers due to their knowledge and understanding of radiology training in their respective countries and can, therefore, be consulted by the ETAP assessors whenever required.

Areas of evaluation:

1. Structure and management of the training programme
2. Delivery of training and education
3. Radiology facilities and resources
4. Research facilities and possibilities
5. Outcomes

The main point of reference for outcomes is the ESR European Training Curriculum for Radiology (ETC) (1) with its five-year training model, consisting of Level I Training over the first three years followed by a Level II Training with special interest rotations during the last two years. It provides both trainees and trainers with a detailed list of learning goals along with the knowledge, skills, competencies, and attitudes that a trainee must acquire during the training programme. Each level has elements that represent learning modules, which mainly consist of organ systems, but they also include components of medical imaging, informatics, management, and radiation protection. Each element is numbered separately and includes the lists of learning objectives for knowledge, skills, competencies, and attitudes. Both the trainees and trainers may utilise each item to investigate the outcome.

### Certification process

Phases of the ETAP 2.0 certification process are:

#### Phase 1: Evaluation and completion of the documentation

**Fig. 2:**
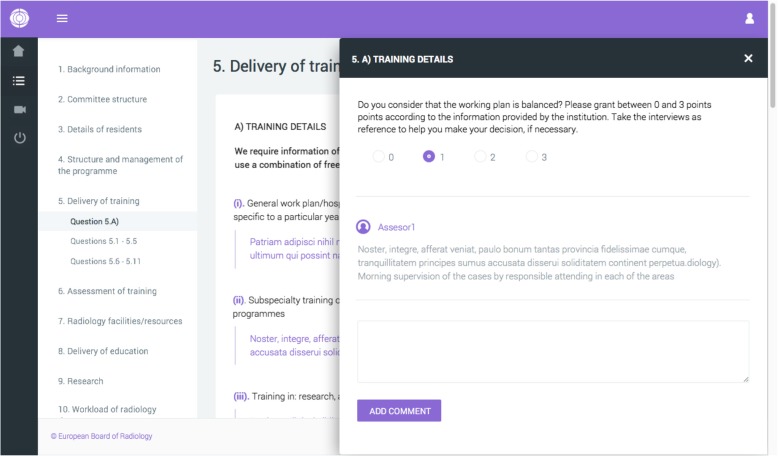
ETAP 2.0 platform (Back-end for assessors)

Centres interested in being assessed submit the application form to the ETAP office using the ETAP platform. The ETAP Scientific Committee verifies compliance with the eligibility criteria and the centre receives its access credentials to the platform to complete the questionnaires. There are two different questionnaires: the main, more extensive questionnaire, to be completed by the head of the training programme, and another one directed to the trainees, to be completed confidentially. Centre and trainees complete their respective questionnaires and provide the necessary information to the assessors (Fig. 2).

#### Phase 2: Video

The centre uploads a video of its facilities and equipment. This verifies the information regarding the facilities and equipment required in the questionnaire, which is filled in by the head of training (report on equipment, including manufacturer, model, year, etc.)

#### Phase 3: Online interviews

Online individual, private and confidential interviews are held with the head or the deputy head of the radiology department, head of the education programme, one of the trainees’ tutors, one attending physician or a deputy involved in the training programme, a trainee supervisor, and at least two trainees (one junior and one senior trainee), according to each institutions’ training department structure.

The assessors and co-assessors conduct online interviews and review the questionnaire, the provided documents and videos in order to evaluate the structure and management of the training programme, the delivery of training and education, the radiology facilities and resources, and the outcomes.

Centres are awarded a certificate according to the assessment performed by the designated assessor and co-assessors. The certificates are valid for a period of 5 years and are subject to renewal.

**Fig. 3:**
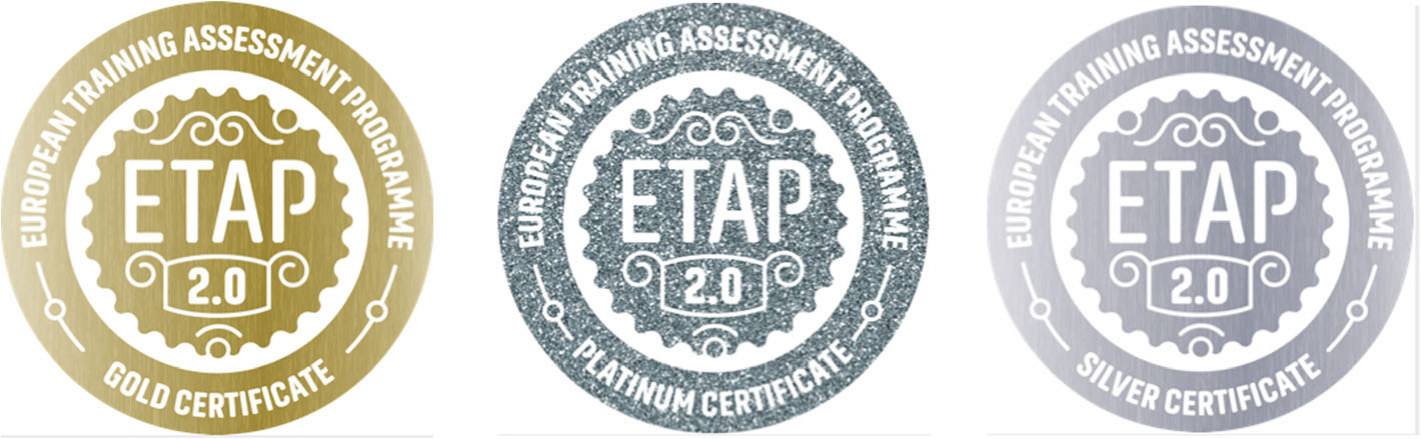
ETAP 2.0 certification ribbons (Gold, Platinum, Silver)

There are three different levels of certification (Fig. 3):

- Silver: the institution has training standards that ensure adequate training in accordance with the standards set out in the ETC and covers all aspects of education.
- Gold: the institution provides a standard of training that is in accordance with the ETC, a subspecialisation programme, and basic research training.
- Platinum: the institution provides an advanced subspecialisation and research training programme and all imaging modalities are available.

**Fig. 4:**
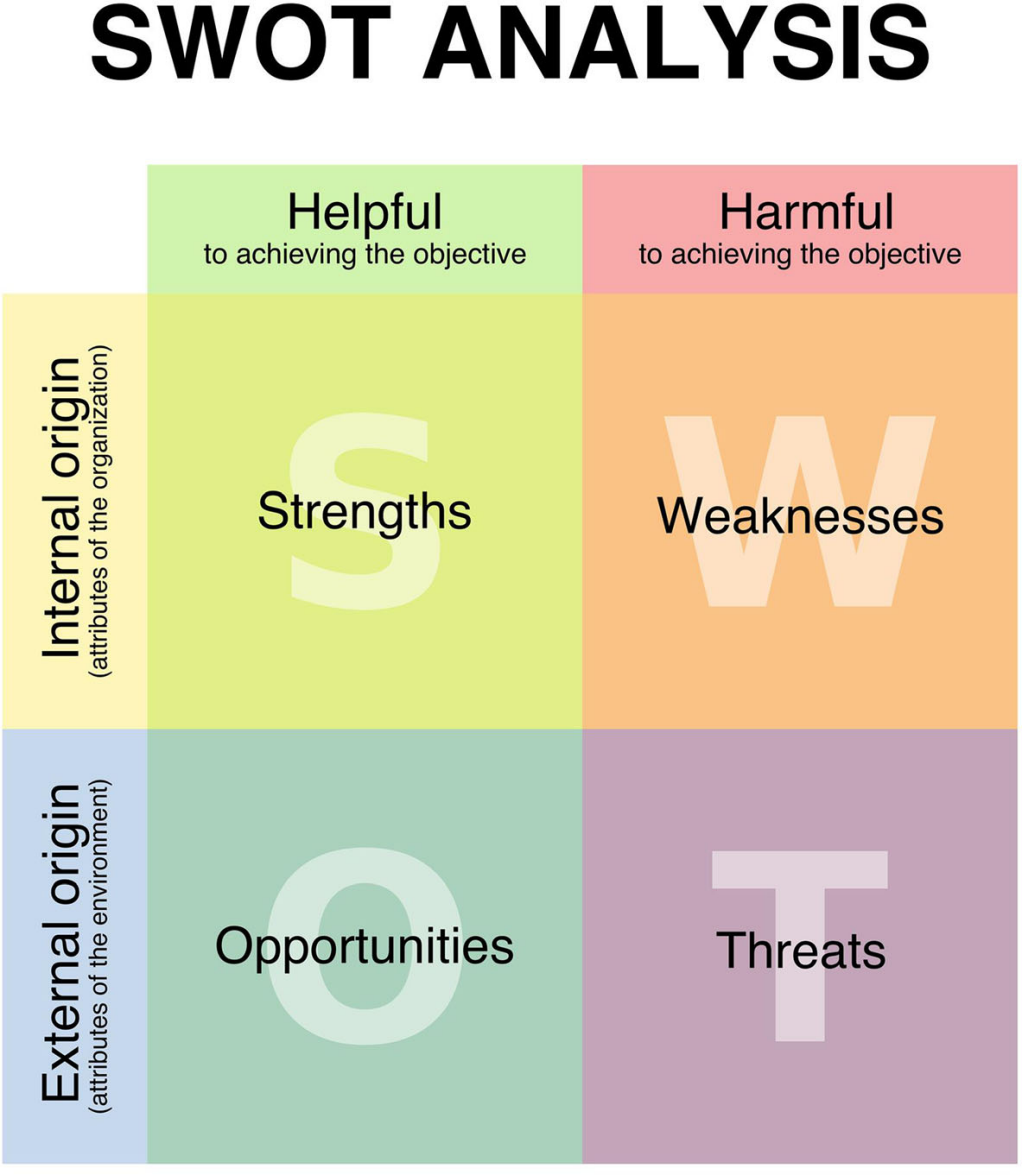
SWOT analysis graphic (Source: Wikimedia Commons)

A precise analysis of the programme is compiled on a structured feedback form and reported back to the centre. The report analyses strengths, weaknesses, opportunities, and threats (SWOT analysis) and, additionally, provides recommendations to help generate further effective strategies to improve the programme (Fig. 4).

After two weeks, the assessed centre is asked to provide initial feedback in the form of an action plan that follows the assessors’ recommendations. Further feedback on the impact of the actions is requested from the training centre after six months.

Four centres have been assessed since the programme was launched in March 2018 (Table 1):

- The University Hospital of Basel (Switzerland): Platinum certificate of excellence
- Kings College of London (United Kingdom): Platinum certificate of excellence
- Hospital Clínic of Barcelona (Spain): Gold certificate of excellence
- Hospital Parc Taulí de Sabadell (Spain): Gold certificate of excellence

**Table 1:** ETAP 2.0 certified centres, number of radiology residents, radiology staff members and number of beds (as of October 23)

Name of the centre the assessed training department belongs to	Number of radiology residents	Number of radiology staff members	Number of beds
University Hospital Basel	22	28	773
Kings’ College Hospital	30	54	1300
Hospital Clínic Barcelona	13	47	819
Consorci Hospitalari Parc Taulí Sabadell (Barcelona)	11	59	700

The assessed institutions had to fill in a feedback form immediately after the assessment. In response to this questionnaire, the institutions stated that the main reasons for applying for ETAP 2.0 certification were to audit their training programme and identify areas of improvement. They also said that they found the certification process objective, transparent, and concise.

According to the feedback received from the certified centres, the SWOT analysis and the recommendations made by the assessors were particularly useful for simulating changes and improving the training programmes.

The ETAP 2.0 strives to cover a wide spectrum of European training institutions and is aware of the heterogeneity that exists within Europe. Therefore, it tries to establish common ground among the different training departments. The advisory programme is essentially based on the content developed by the ETC [1], which establishes the basis for creating a homogeneous training system throughout all European centres. This, in turn, facilitates the creation of a common and interchangeable certification and aids the mobility of professionals within the European countries.

Training centres from many European countries have been assessed since 2001. What sets the ETAP 2.0 apart is its platform which enables a quick and easy certification process for both applicants and assessors. Both, representatives of the applicant institutions and the assessors can easily and efficiently store, access, and manage all documents and information necessary for the certification process.

Accuracy and objectivity are guaranteed thanks to the detailed questionnaire which must be completed by the centre and verified during the online interviews with the chairperson of the department, the head of training, and the residents. The work carried out before the online evaluation interviews is very useful for an adequate assessment and for providing the best advice to the centres.

Every radiology training programme in Europe could be improved. ETAP 2.0 offers training centres an opportunity for self-development [2]. Self-evaluation of the training programme is essential for auditing purposes.

Although radiological research is not the main aspect evaluated by ETAP 2.0, the training centre will not be awarded the maximum score if it does not support and encourage its trainees to conduct research. An active research programme aimed at younger residents represents an essential part of Level III and Level IV training and the evaluation of this aspect is a crucial part of the ETAP 2.0 process.

The requirements for the European Training curriculum do not only cover the knowledge, but also skills and attitudes which are nowadays increasingly important in the healthcare field. These aspects are also considered in the assessment.

The final score of the assessed training centres is determined using a weighting system for the different areas of evaluation based on the information provided by the institution; namely the questionnaires and information provided by the head of training and the residents, the video of the institution facilities and equipment, and the online interviews. The weighting of the questionnaire is based on the relevance of each question with regard to the skills, knowledge, competences, and attitudes that a trainee must acquire during the training programme.

Qualitative and objective feedback from the assessors is an essential part of the assessment process [2, 3]. The accessibility as well as the quality of the questionnaires available on the webpage of ETAP 2.0 aid the training programmes and, at the same time, enhance advisory process [2].

The ETAP 2.0 certification, classified in three levels (Silver, Gold, and Platinum), guarantees that the training department meets the quality standards set by the European Society of Radiology (ESR European Training Curriculum (ETC)) and the UEMS, providing it with European and international recognition. It further certifies that the training programme is effective and validates the residents’ level of competence, attitude, and development of new skills. The expectations and recommendations are compatible with the ESR ETC (1) and modern international educational references [4].

The certificate is valid for five years, which is the period set for all centres to maintain and improve their degree of excellence in accordance with the ETC, following the recommendations of the assessment. ETAP 2.0 offers a cost-effective way to accomplish this task. We, as radiologists, evaluate our specialised training centres and protect the autonomy of radiology by maintaining high standards of excellence.

Training centres may have different reasons for obtaining the certification. Accepting the audit process as a self-assessment and quality assurance tool could be one of the main motivations. Institutions may also use the feedback showcasing their weaknesses as an opportunity to improve their training. This feedback may also be useful to provide a rationale for the administrations of the centres, which we believe will result in a positive outcome.

## Summary

Terms of legislation and training programmes across Europe vary. The harmonisation of training programmes will support the recognition of common certification and licensing throughout Europe and improve quality of care and patient safety. ETAP 2.0 is an excellent quality control tool that provides a precise analysis and an objective outside perspective. It represents an instrument which can standardise and facilitate accreditation across the board as well as promote the mobility of professionals, expand horizons, and help enrich and enhance the profession.

## Data Availability

All data generated or analysed during this study are included in this published article.
